# Getting a hold on archaeal type IV pili: an expanding repertoire of cellular appendages implicates complex regulation and diverse functions

**DOI:** 10.3389/fmicb.2015.00362

**Published:** 2015-05-05

**Authors:** Scott Chimileski, R. Thane Papke

**Affiliations:** Department of Molecular and Cell Biology, University of ConnecticutStorrs, CT, USA

**Keywords:** haloarchaea, type IV pili, archaellum, surface adhesion, archaeal biofilm formation, microbial cell-to-cell interactions

Type IV pili (T4P) are a group of cell surface appendages of particular interest due to broad conservation and functional versatility across the domains *Bacteria* and *Archaea* (Albers and Meyer, 2011; Giltner et al., 2012). All T4P are composed of small protein subunits known as pilins that polymerize into helical fibers through the action of assembly ATPases (Giltner et al., 2012). This core ancestral machinery has been adapted in various lineages for many cellular processes–from adhesion and biofilm formation, to motility, horizontal gene transfer (HGT) and even electricity conduction (Giltner et al., 2012; Berry and Pelicic, 2015). When T4P structures are involved in adhesion, they are known as pili, if they no longer mediate attachment, but are associated with another function, such as scavenging macromolecules (e.g., DNA uptake by Com proteins in *Bacillus subtilis*), or secretion of proteins through a piston-like structure (i.e., type II secretion), they are called pseudopili (Averhoff and Friedrich, 2003; Peabody et al., 2003; Chen et al., 2005). T4P appendages may also contribute to both adhesion and another function. This dual function is sometimes true for archaella: a major group of archaeal T4P appendages characterized by the ability to rotate and enable swimming motility. Archaella are functionally analogous yet structurally and genetically unrelated to bacterial flagella (Jarrell and Albers, 2012; Shahapure et al., 2014; Albers and Jarrell, 2015). T4P have been studied to a greater extent in bacteria, in part because they are often virulence factors (Giltner et al., 2012). However, recent investigations have revealed a repertoire of archaeal T4P–highlighting implications for regulatory complexity and functional diversity.

Losensky et al. (2014) demonstrated that adhesive filaments in the haloarchaeon *Halobacterium salinarum* R1 observed during biofilm formation (Fröls et al., 2012) are dependent on the pilus assembly ATPase gene *pilB1* (Losensky et al., 2014), expanding the list of experimentally studied archaeal T4P (Table 1). Deletion of *pilB1* led to a lack of pili as observed through electron microscopy and a defect in adhesion. Only 4% of a glass surface was colonized by non-piliated/non-archaellated cells (Δ*flaI*/Δ*pilB*1), relative to 36 and 44% for the parental and non-archaellated (Δ*flaI*) strains, respectively. The molecular composition of PilB1-dependent pili has not yet been determined, however Losensky and coauthors noted that there are over 30 candidate pilins in the *Hbt. salinarum* R1 genome, as indicated by the class III signal peptide prediction program FlaFind (Szabó et al., 2007b). FlaFind was used previously to show that most archaeal genomes contain many pilin/archaellin homologs (Szabó et al., 2007b; Esquivel et al., 2013). For example, *Haloarcula marismortui* and *Haloferax volcanii* have nearly 50 putative pilin/archaellin precursors (Esquivel et al., 2013).

**Table 1:** **Experimentally studied type IV pili in archaeal species: archaella, adhesive pili, and pseudopili^a^**.

**Structure name**	**Function/associated phenotype**	**Characterized in (genera)**	**Filament diameter (nm)**	**References**
**ARCHAELLA**				
	Swimming motility and involved in adhesion in some species; functionally analogous but evolutionarily and structurally distinct from the bacterial flagella^b^	*Halobacterium*	10–15	Alam and Oesterhelt, 1984; Gerl and Sumper, 1988; Patenge et al., 2001; Streif et al., 2008
		*Haloferax*		Tripepi et al., 2010, 2012, 2013
		*Haloquadratum*		Alam et al., 1984
		*Sulfolobus*		Szabó et al., 2007a; Lassak et al., 2012b; Shahapure et al., 2014
		*Methanococcus*		Bardy et al., 2002; Jarrell et al., 2011
		*Methanocaldococcus*		Bellack et al., 2011
		*Pyrococcus*		Nather et al., 2006; Nather-Schindler et al., 2014
**ADHESIVE PILI**				
Archaeal adhesive pilus (Aap)	Surface adhesion	*Sulfolobus*	11	Henche et al., 2012
Type IV pilus (Epd)	Surface adhesion	*Methanococcus*	8.5	Vandyke et al., 2008; Wang et al., 2008; Nair et al., 2013
Type IV pilus (PilA)	Surface adhesion	*Haloferax*	8–12	Esquivel et al., 2013; Esquivel and Pohlschröder, 2014, 2015
PilB1-dependent adhesive pilus-like^c^	Surface adhesion	*Halobacterium*	7–8	Losensky et al., 2014
UV-inducible pilus (Ups)	Autoaggregation and species specific DNA exchange following UV-irradiation	*Sulfolobus*	10	Fröls et al., 2008; Ajon et al., 2011
**PSEUDOPILUS-LIKE**				
Bindosome assembly system (Bas)	Sugar binding; also involved in cellular morphology and S-layer architecture	*Sulfolobus*	ND	Zolghadr et al., 2007, 2011

*ND, not determined*.

a *As reviewed by Pohlschröder et al. (2011), Lassak et al. (2012a), Jarrell et al. (2013) *and* Esquivel and Pohlschröder (2015)*.

b *See Jarrell and Albers (2012), Shahapure et al. (2014) and Albers and Jarrell (2015) for a review of how the archaellum came to be recognized as a unique archaeal motility structure, including a more comprehensive categorization of characterized archaella*.

c *The appendages characterized by Losensky et al. (2014) are listed as pilus-like because they have yet to be purified and biochemically verified. They may in fact be composed of homologs of PilA pilin proteins in H. volcanii, which are found in many euryarchaeal species (Esquivel et al., 2013)*.

Some of these pilins could be associated with additional functions. *Hfx. volcanii* has an ability for social motility in static liquid (Chimileski et al., 2014) and T4P could be involved in this activity (Esquivel and Pohlschröder, 2015), whereby they may attach to extracellular matrix along the substratum, similar to the S-motility system that pulls *Myxococcus xanthus* cells forward (Hodgkin and Kaiser, 1979; Zusman et al., 2007). There could be more archaeal T4P-related surface structures that scavenge macromolecules as well, like the bindosome of *Sulfolobus solfataricus* (Zolghadr et al., 2007, 2011).

Investigations of archaeal T4P leave open the possibility for undiscovered mechanisms for contacting abiotic surfaces or other cells. For instance, even in the non-piliated/non-archaellated *Hbt. salinarum* strain, adhesion was not completely abolished (Losensky et al., 2014). Similar residual adhesion has been observed in *Hfx. volcanii* (Tripepi et al., 2010, 2013). In both cases, pilins that remain present in the membrane but cannot be assembled into pili without the assembly ATPase(s) likely explain low levels of adhesion (Esquivel and Pohlschröder, 2014). There are two other cell-to-cell contact phenomena in *Hfx. volcanii* that do not require archaella or pili: Ca^2+^ dependent autoaggregation (Tripepi et al., 2010), and an HGT mechanism known as mating (Rosenshine et al., 1989; Tripepi et al., 2010; Naor et al., 2012). Additional types of extracellular polymers or fibers found in bacterial species could be present in archaea, such as amyloid protein (Chimileski et al., 2014). Unusual, genetically ambiguous non-T4P structures have already been observed in other archaeal species, including the hamus of the SM1 euryarchaeon (Moissl et al., 2005; Perras et al., 2014) and the cannulae of *Pyrodictium* cells (Nickell et al., 2003).

A plausible explanation for having a wide array of appendages is a capacity for differential regulation (Jarrell, 2012; Lassak et al., 2012a; Jarrell et al., 2013). Indeed, a number of studies point to dynamic regulatory systems controlling archaeal T4P. In *Hbt. salinarum, pilB1* expression was upregulated relative to *flaI* in adherent cells (Losensky et al., 2014), suggesting archaella and pili have antagonistically regulated functions in motility (when a planktonic state is favorable) and for adhesion (during biofilm formation), as in *Hfx. volcanii* (Tripepi et al., 2010; Esquivel and Pohlschröder, 2014, 2015). *Haloarcula marismortui* has two archaellins that are expressed under different temperatures and salinities (Syutkin et al., 2014), termed ecoparalogs. Likewise, the six *Hfx. volcanii pilA* paralogs, any one of which can restore adhesion when expressed in a null mutant [Δ*pilA(1–6)*] (Esquivel et al., 2013), may be ecoparalogs as well. Intriguingly, deleting *flgA2*, one of two archaellin genes in *Hfx. volcanii*, produced a hypermotile phenotype, rather than a motility defect (Tripepi et al., 2013). Archaellins are also regulated through N-glycosylation (Guan et al., 2012; Tripepi et al., 2012) and regulatory proteins controlling adhesive pili and archaella have been identified in *Sulfolobus acidocaldarius* (Reimann et al., 2012; Orell et al., 2013; Vassart et al., 2013).

As more T4P are described in archaeal groups, a common theme is appearing. A multitude of individual pilins/archaellins from one or more loci may appear to be redundant–contributing to appendages that are difficult to differentiate through electron microscopy and often depend on the same assembly ATPase. However, to the contrary, the maintenance of more than one pilus and archaellum subunit gene is likely due to a complex regulatory network and the corresponding advantages of functional versatility. Subsets of pilins may be expressed in different combinations as a response to a variety of specific environmental conditions and/or cellular functions.

## Conflict of interest statement

The authors declare that the research was conducted in the absence of any commercial or financial relationships that could be construed as a potential conflict of interest.
